# Risk factors for rod fracture after posterior correction of adult spinal deformity with osteotomy: a retrospective case-series

**DOI:** 10.1186/s13013-015-0056-5

**Published:** 2015-11-04

**Authors:** Cameron Barton, Andriy Noshchenko, Vikas Patel, Christopher Cain, Christopher Kleck, Evalina Burger

**Affiliations:** Department of Orthopedics, University of Colorado, Anschutz Medical Campus, 12631 E. 17th Avenue B202, Aurora, CO 80045 USA

**Keywords:** Adult spinal deformity, Osteotomy, Rod fracture, Risk factors

## Abstract

**Background:**

Osteotomies including pedicle subtraction (PSO) and/or Smith-Peterson (SPO) are used to facilitate surgical correction of adult spinal deformity (ASD), but are associated with complications including instrumentation failure and rod fracture (RF). The purpose of this study was to determine incidence and risk factors for RF, including a clinically significant subset (CSRF), after osteotomy for ASD.

**Methods:**

A retrospective review of clinical records was conducted on consecutive ASD patients treated with posterolateral instrumented fusion and osteotomy. Seventy-five patients (50 female; average age, 59) met strict inclusion/exclusion criteria and follow-up of ≥1 year. Data was extracted pertaining to the following variables: patient demographics; details of surgical intervention; instrumentation; and postoperative outcomes. Patients were divided into two subgroups: 1) rod fracture (RF) and 2) non-RF. The RF subgroup was further divided into CSRF and non-CSRF. Odds ratios (OR) were calculated to evaluate the association between risk factors and RF. The *χ*^2^-test was used to define *P*-values for categorical variables, and *T*-test was applied for continuous variables, *P*-values ≤0.05 were considered significant.

**Results:**

Incidence rates of RF were: for entire population, 9.3 % (95 % Cl: 2.7 %; 15.9 %); for PSO, 16.2 % (95 % Cl: 4.3; 28.1); and for SPO, 2.6 % (95 % Cl: 0 %; 7.7 %); the OR of PSO versus SPO was 7.2 (95 % Cl: 0.8; 62.7, *P* = 0.1). CSRF incidence was 5.3 % (95 % CI: 0.2 %; 10.4 %). Significant risk of RF was revealed for following factors: fusion construct crossing both thoracolumbar and lumbosacral junctions (*OR* = 9.1, *P* = 0.05), sagittal rod contour >60° (*OR* = 10.0, *P* = 0.04); the presence of dominos and/or parallel connectors at date of rod fracture (*OR* = 10.0, *P* = 0.01); and pseudarthrosis at ≥1 year follow-up (*OR* = 28.9, *P* < 0.001). Statistically significant risk of CSRF was revealed for fusion to pelvis (*P* = 0.05) and pseudarthrosis at ≥1 year follow-up (*OR* = 50.3, CI: 4.2; 598.8, *P* < 0.01).

**Conclusions:**

The risk of RF after posterolateral instrumented correction of ASD with osteotomy had statistically significant association with the following factors: pseudarthrosis at ≥1 year follow-up; sagittal rod contour >60°; presence of dominos and/or parallel connectors at date of fracture; and fusion construct crossing both thoracolumbar and lumbosacral junctions. Statistically significant risk for the CSRF subset was fusion to the pelvis and pseudarthrosis at ≥1 year follow-up.

## Background

Adult spinal deformity (ASD) is a debilitating condition that often requires surgical correction. The aging population, combined with an increasing number of spine fusion procedures, is increasing the prevalence of ASD [1, 2]. Osteotomy procedures, including Smith-Peterson (SPO) and pedicle subtraction (PSO) are effective procedures used to correct deformity and lead to improved clinical outcomes [3–6]. One important difference between these osteotomies is that SPOs only involve resection of the posterior column of the spine while PSOs involve a greater resection including the pedicle and wedge of vertebral body [7, 8]. As a result, one PSO can achieve greater correction (30–40°) than a single SPO (approximately 10°) [9–13]. Due to the physical differences in osteotomies, it would seem plausible that after PSO vs. SPO the spine and fusion construct would be subjected to different strains and subsequent complications. However, while mechanical complications after PSO have been well described, these complications after SPO haven’t specifically been of focus. Multiple authors have described increased complications [4, 6, 14–16], pseudarthrosis [3, 4, 14, 15, 17], and instrumentation failure including rod fracture (RF) in PSO patients [4, 6, 14, 18]. Of note, rod fracture often requires reoperation and studies have postulated a link between pseudarthrosis and RF [4, 6, 14, 18]. However, prior studies have not statistically confirmed this linkage due to study limitations.

Rod fracture can be differentiated into two main subgroups: clinically significant rod fracture (CSRF) and non-CSRF (CSRF was termed “symptomatic rod fracture” in a previous study) [18]. CSRF can be defined as symptoms prompting evaluation (e.g., pain, neurological symptoms) and diagnosis of fractured rods via radiographic imaging or re-operation. CSRFs are often accompanied with radiographic signs of pseudarthrosis, new symptoms such as pain or radiculopathy, or resulting in loss of correction [18], while non-CSRFs by definition aren’t associated with patient symptoms or loss of correction. Previous studies have found that the incidence of RF varies widely after spinal correction, with a recent study citing a post-PSO incidence of 22 % for all RF including non-CSRF and CSRF [4, 14, 18, 19]. The largest study to date studying CSRF only, reported a lower incidence rate of 6.8 % in a population of ASD patients treated with long, >5 level posterior instrumented fusion, with a higher incidence in a subset of patients who underwent osteotomy at 15.8 % [18]. Of note, no large series to date has cited the incidence of RF and CSRF subset in a combined cohort of adult spinal deformity patients treated with either PSO and/or SPO and compared the incidence between the two procedures.

Prior authors have suggested subdividing the etiology of RF into two main categories: early and late RF [18]. Early RF (<12 months) is thought to be attributed to primary instrumentation failure and may lead to delayed union while late RF (>12 months) may be partially attributed to pseudarthrosis [18].

Biomechanical risk factors for rod fracture studied to date can be divided into two main categories: intrinsic and extrinsic properties. Intrinsic properties include material type (mainly stainless steel, titanium, and cobalt chromium alloys) and diameter (usually ranging from 5.5–6.35 mm). Pertaining to metal type and rod diameter, studies have either found no statistically significant differences or have published conflicting results [18, 19]. Extrinsic factors can be divided into several previously described biomechanical notions. The first notion states that primary contouring or metal bending (including the use of French benders) has shown to weaken metal and decrease fatigue strength [20, 21]. Further, repeated bending or rod contouring to an extreme angle has been hypothesized to decrease rod fatigue strength [20–25]. While biomechanical studies have shown a link between “excessive contouring” and decreased fatigue strength, no clinical study to date has confirmed if this “excessive contouring” poses a significant risk in real-world surgical applications. Additionally, “notch sensitivity,” results from defects in the surface of metal, particularly titanium, which decrease the strength of the metal [26, 27]. Third, cyclic loading causes repeated metal strain leading to inherent metal fatigue and failure, particularly in titanium constructs [22–24, 28], while single incident instrumentation overloading is less common [28]. Similarly, notch sensitivity and cyclic loading haven’t received statistical confirmation in clinical studies.

Several risk factors have been confirmed statistically in clinical applications. A recent study on RF in patients undergoing instrumented posterior fusion (including a subset of PSO patients) found statistically significant risk factors for RF in this population including age, body mass index (BMI), baseline sagittal imbalance, baseline pelvic incidence minus lumbar lordosis (PI-LL) mismatch, and greater sagittal imbalance correction [19]. This study found no statistically significant risk for RF pertaining to smoking, levels fused, and rod diameter. Further, the literature includes a limited number of clinical studies that have proposed risk factors for mechanical complications (some encompassing RF) following posterior spinal fusion in various populations. Some of these variables are thought to have an association with instrumentation failure by increasing construct strain and include under-corrected sagittal vertical axis (SVA) (*OR* = 17.5) [29], number of instrumented vertebra (*OR* = 2.23–11.46 depending on number of levels) [14], and fusion across transitional spine junctions such as the lumbosacral junction (*OR* = 2.8) [14, 30]. Other variables linked to mechanical complications include absence of anterior column support (i.e., interbody fusion), insufficient distal foundation, and fusion to the sacrum [16, 18, 30–32]. However, these studies often included a broad population of instrumented posterior fusion, did not analyze RF and CSRF specifically, and did not provide risk assessment analysis for osteotomy patients. Therefore, analyzing risk factors for RF, specifically CSRF after osteotomy is important. The purpose of this study was to determine incidence and risk factors for RF and CSRF after posterior instrumented correction of adult spinal deformity with osteotomy.

## Methods

### Patient population

**Fig. 1:**
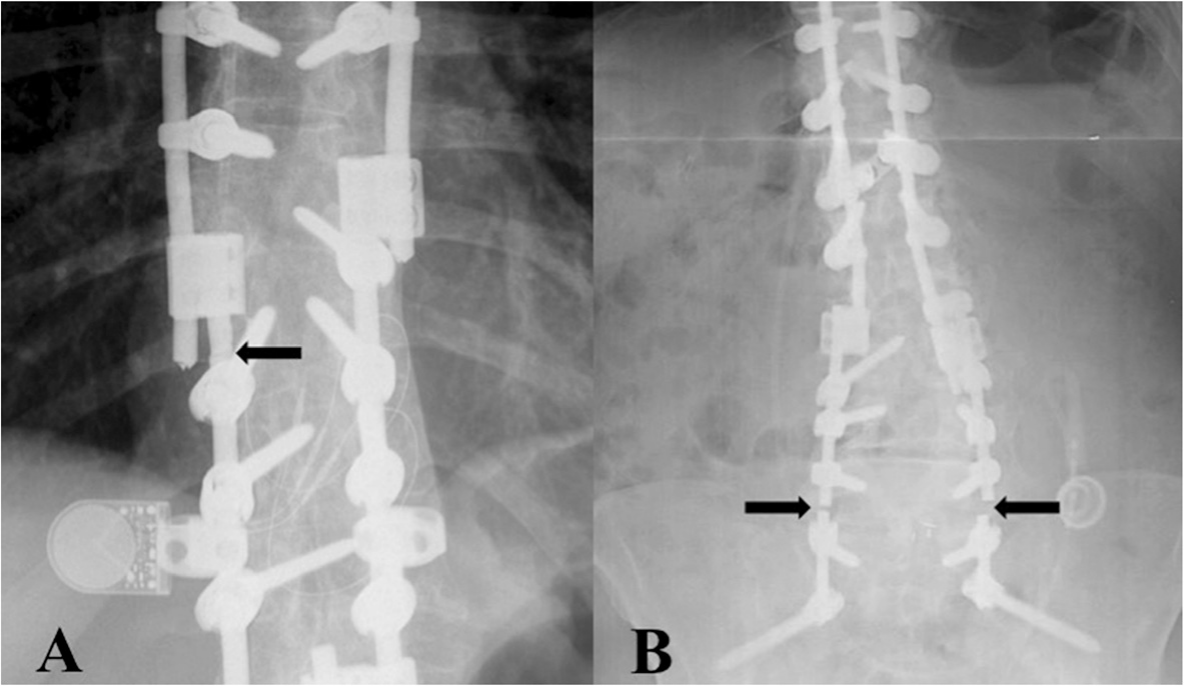
Radiographic findings of rod fracture. **a** Right T10 rod fracture near domino connector (rod fracture #6) diagnosed 58 months postop with associated T7-8 pseudarthrosis. This fracture occurred at location of prior cross-link. This patient had two subsequent PSO operations after the original PSO operation. The rod fracture occurred in a rod from the first PSO operation. Dominos were added during a subsequent PSO operation to connect to original instrumentation. **b** Bilateral L5-S1 rod fractures (rod fractures #1 and #2) diagnosed at 12 and 20 months post-operation with associated L5-S1 pseudarthrosis. These rods fractured at the apex of the rod bend with an 81° sagittal rod contour. Pseudarthrosis at L5-S1 was diagnosed during the second rod fracture at 20 months post-operation

After institutional review board approval (COMIRB #14-1258), data was analyzed from 104 consecutive patients who underwent an instrumented posterolateral spinal fusion including a SPO, PSO, or combination for adult spinal deformity between 2007 and 2014 by 4 surgeons at a single institution. Inclusion criteria consisted of: 1) >18 years of age; 2) diagnosis of adult spinal deformity including the following etiologies: fixed sagittal imbalance, idiopathic and degenerative scoliosis or kyphosis, posttraumatic kyphosis, idiopathic flat back syndrome, and ankylosing spondylitis; 3) operation consisting of ≥2 level instrumented posterolateral fusion with lumbar or thoracic spinal osteotomy (PSO and/or SPO) with or without interbody fusion for spinal deformity; and 4) follow-up for up to 7 years. Exclusion criteria consisted of: 1) osteotomy procedure for other diseases such as: tumor or infection; and 2) latest follow-up <1 year unless re-operation for rod fracture 3) secondary operation with manipulation of instrumentation during first postoperative year, for reasons other than rod fracture (e.g., infection with primary instrumentation removal or exchange). These patients were excluded from risk factor analysis because their variables couldn’t serve as a uniform comparison against cases of RF. This thought was based on the possibility that the original rods could have been replaced or subject to different additional stressors (e.g., addition of iliac bolts during subsequent operation) than were primarily documented. Further, radiographic detection of callus ossification after fusion surgery typically requires 9–12 months. Thus, it could impact evaluation of a risk associated with pseudarthrosis. CSRF was defined as a combination of symptoms prompting evaluation (as described below) and radiographic and/or intraoperative evaluation, if re-operation was completed. The main clinical criteria included: new symptoms such as pain, neurological symptoms, prominence of instrumentation, or worsening of spinal deformity or loss of correction. Other symptoms prompting evaluation included an audible or tactile “pop.” Radiographic diagnosis included rod irregularities suggesting a crack or discontinuation of rod contour (Fig. 1: a; b), particularly if accompanied by radiographic suspicion of pseudarthrosis. Pseudarthrosis was initially diagnosed via helical CT with sagittal and coronal reconstruction and confirmed intra-operatively via detection of spine movement between motion segments within the fusion mass.

### Data collection

**Fig. 2:**
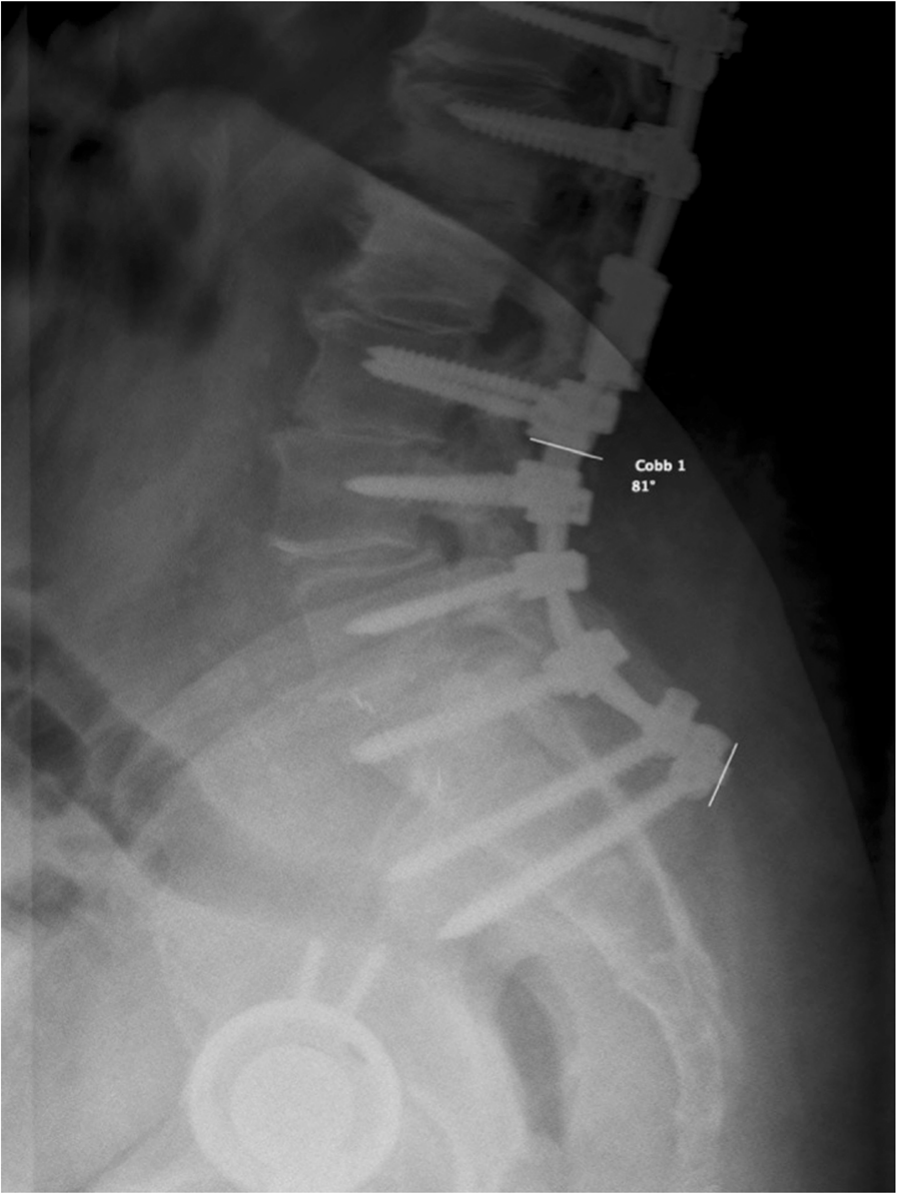
Measuring sagittal rod contour. A Cobb angle was drawn on lateral radiographs incorporating the entire rod curvature. Both lines were drawn perpendicular to the rods

The patients’ medical records were reviewed and the following data was extracted and divided into four main categories:

1. Patient variables: demographics (age, gender, ethnicity), and patient baseline characteristics (BMI, comorbidities, smoking status, primary diagnosis, primary spine surgery vs. repeat, presence of pseudarthrosis at primary osteotomy operation);
2. Surgical variables: number of posterior levels fused, location of fused levels, fusion to sacrum, pelvic fixation, osteotomy type (SPO or PSO), and osteotomy location. It should be noted that all patients received bone grafting posteriorly, and were classified as posterolateral fusions. The specific type of bone grafting was not analyzed;
3. Instrumentation variables: manufacturer; screws, polyaxial or monoaxial; rods, precontoured or straight; rod material; rod diameter; pedicle screw density, described as complete or incomplete – missing one or more pedicle screws at available locations along construct; type of rod connectors, standard vs. all others including de-rotation connectors; presence of interbody support - including interbody allograft/autograft, titanium or PEEK cages, anterior plating, axial lumbar interbody fusion, and/or lateral mass screws postoperatively; sagittal rod contour angle; location of apex of rod bend; crosslink and domino/parallel-connector number and location. It should be noted that multiple patients had presence of interbody fusion before the osteotomy operation, with incomplete records with regards to the type of interbody device and bone graft. Therefore, we analyzed the risk for rod fracture of presence of interbody fusion in immediate post-operative imaging. Absence of pedicle screws at PSO location was still considered “complete,” as pedicle screws cannot be placed at PSO level. Sagittal rod contour angle was measured on sagittal radiographs, using a Cobb angle encompassing the total rod curve (Fig. 2); 4) Postoperative variables: spinopelvic parameters (sagittal vertical axis (SVA), coronal balance, lumbar lordosis (LL), thoracic kyphosis (TK), pelvic tilt (PT), pelvic incidence (PI), coronal cobb); subsequent surgeries; presence of rod fracture and type (CSRF vs. non-CSRF) in follow-up period; presence of pseudarthrosis in follow-up period; history of a fall or other trauma in follow-up period; reoperation in follow-up period; and total follow-up duration.

### Incidence analysis

All patients meeting inclusion and exclusion criteria were included for RF and CSRF incidence analysis. Patients were then divided into SPO and PSO subgroups to determine incidence by osteotomy type. If patients had both SPOs and PSO, they were included in the PSO group only.

### Risk factor analysis

Patients were divided into two subgroups based on rod fracture alone: RF vs non-RF (i.e., patients not experiencing a rod fracture in follow-up period). Rod fracture patients were further divided into CSRF vs. non-CSRF. Variables listed above were compared between subgroups and significant differences were noted. Each case of RF was additionally analyzed taking into consideration such specific factors as: location of RF, in particular relative fusion level; location of screw/connector as potential cause of notch effect; and documented repeated contouring during a subsequent operation via in-situ benders (e.g., the ends of primary implanted rods may need to be re-contoured to connect to new instrumentation).

Radiographic characteristics listed above were measured with facilitation of Surgimap surgical planning software (Nemaris Inc, New York, NY) on previously obtained longstanding scoliosis x-rays.

### Statistical methods

Incidence rate was defined by percentage with 95 % confidence limits (95 % Cl). Risk assessment was performed by odds ratio (OR) with 95 % Cl, *P*-value was defined by *χ*^2^-test. To compare continuous variables, *T*-test was used, P- values ≤0.05 were considered statistically significant [33].

## Results

### Total patient population

In total, 104 patients met initial inclusion criteria. Twenty patients were excluded from the study based on greatest follow-up <1 year. Additionally, 9 patients were excluded based on a subsequent operation during first postoperative year for reasons other than rod fracture. Seventy-five patients were left for analysis including 50 female and 25 male, mean age was 59 (range, 24–82; SD, 12.9), Table 1. Mean follow-up for entire group was 32 months. By subgroup, mean non-RF follow-up was 31 months (range 11–75 months) and mean RF follow-up was 41 months (range 14–64 months) with average time to rod fracture 20 months (range 11–58 months). Seventeen of 75 patients (23 %) did not reach an approximate 2-year follow-up; defined as clinical visit, imaging, or follow-up correspondence at ≥20 months. Eleven of 17 patients were < 2 years post-operation (obtaining a range of follow-up from 12–20 months) while the remaining 6 of 17 were considered true lost to follow-up (LTFU). Of the 6 LTFU patients, 4 were unable to be reached via follow-up phone calls and 2 had deceased.

**Table 1:** Demographics of risk factor analysis group, *N* = 75

Characteristics	Index	Value
Age	Mean (St. D)	58.9 (12.9)
Gender: Female	N (%)	50 (66.6 %)
Male	N (%)	25 (33.3 %)
Ethnicity: Caucasian	N (%)	66 (88.0 %)
Hispanic	N (%)	4 (5.3 %)
Refused	N (%)	5 (6.7 %)
Previous spinal surgical intervention:	N (%)	16 (21.3 %)
Primary		
Reoperation	N (%)	59 (78.7 %)
Body mass index (BMI)	Mean (St. D)	26.7 (5.5)
Smoking status: Never smoker	N (%)	31 (41.3 %)
Ever smoker	N (%)	36 (48.0 %)
Not specified	N (%)	8 (10.7 %)

### Incidence analysis

In our case-series, incidence of RF was 9.3 % (95 % Cl:2.7 %; 15.9 %) with 7/75 patients experiencing fractures. By subtype 4/7 were classified as CSRF, resulting in CSRF incidence of 5.3 % (9 5% CI: 0.2 %; 10.4 %). When separated by osteotomy type, the RF incidence rate trended higher if operation included PSO (6/37, 16.2 %, 95 % Cl: 4.3 %; 28.1 %) versus SPO only (1/38, 2.6 %, 95 % Cl: 0 %; 7.7 %), but this difference was found to have borderline significance (*OR* = 7.2 95 % CI: 0.8; 62.7, *P* = 0.1). When CSRFs were separated by osteotomy type, the CSRF trended higher if operation included PSO (3/37, 8.1 %, 95 % CI: 0 %; 16.9 %) versus SPO only (1/38, 2.6 %, 95 % CI: 0 %; 7.7 %). The 3 non-CSRFs were not associated with symptoms, pseudarthrosis, and didn’t require re-operation. Six of 7 patients experienced unilateral rod fracture and 1 patient experienced bilateral rod fractures.

#### Description of clinically significant rod fracture cases

After assessment of individual broken rods (5 total), CSRF patients held several notable characteristics. No CSRFs were found <12 months postop with an average time to fracture of 14.8 months. Rod fractures #1 and #2 were diagnosed 12 and 20 months postop and occurred in the same patient at the L5-S1 level with associated L5-S1 pseudarthrosis, Fig. 1b. These rods fractured at the apex of the rod bend with an 81° sagittal rod contour. Pseudarthrosis at L5-S1 was diagnosed during the second rod fracture at 20 months. Rod fracture #3 was diagnosed 14 months postop and occurred at T11-T12 with associated T11-12 pseudarthrosis. This fracture occurred near the level of a domino and level of T11 PSO. Rod fracture #4 was diagnosed 12 months postop and occurred at the iliac bolt connector. During a revision operation post original osteotomy, this same rod was noted to be “bent out of the way” and subsequently bent back to connect to iliac bolt connector. This break occurred near the apex of rod bend occurring at S1 with a sagittal bend of 66°. Rod fracture #5 was diagnosed at 16 months postop and occurred above the iliac bolt connector and was associated with a patient fall and pseudarthrosis at L4-5 and L5-S1.

#### Description of non-clinically significant rod fracture cases

Three non-CSRFs were observed in this study. Rod fracture #6 occurred at T10, near the level of a domino and location of prior cross-link Fig. 1a. This patient had two subsequent PSO operations after the original PSO operation, with rod fracture occurring in the original rod from the first PSO operation. This fracture was diagnosed after all PSO operations at 58 months postop after the patient felt a “pop” and shoulder pain. Follow-up CT scans showed solid osseous fusion across the entire construct. Shoulder MRI provided evidence that pain was likely from rotator cuff tendinosis and labral tear. Of note, immediate postoperative films showed 2 cm SVA that increased to 10 cm before second PSO and remained at 10 cm before third PSO. Domino connectors were added during the subsequent PSOs to connect to original instrumentation. Rod fracture #7 occurred at L3-4, near apex of 54° sagittal rod contour and L4 PSO, and was detected via follow-up radiographs at 11 months post-operation. CT scans showed solid osseous fusion across the entire construct. No symptoms other than an initial “pop” were described in this patient. Rod fracture #8 was detected via follow-up phone call in which patient was able to explain his L3 rod fracture diagnosis and lack of clinically significant findings. L3 was near apex of 66° sagittal rod contour and location of L3 PSO. However, no rod fracture follow-up records were obtained from this patient and exact time of fracture is unclear.

### Risk factors analysis: all rod fractures

Demographic characteristics such as: age, gender, ethnicity, BMI, previous spine surgery, or smoking status did not show significant association with RF risk, Table 2. No apparent trends were noted in diagnosis subclass or type and number of comorbidities.

**Table 2:** Association of rod fracture risk with demographic characteristics

Confounders	Subgroups	Rod(s) fracture		Odds ratio (95 % Cl: min; max)	P(*χ* ^2^)
		Yes	No		
Gender	Female	3	47	0.3 (0.07; 1.6)	0.2
	Male	4	21		
Age	24–59 years	2	33		
	60–82 years	5	35	0.4 (0.08; 2.3)	0.3
Ethnicity	Caucasian	6	60	N/A	>0.5
	Hispanic	0	4		
	Refused	1	4		
Body mass index (BMI)	>30	2	14	1.2 (0.2; 6.7)	>0.5
	≤30	5	41		
Previous thoracolumbar surgery	Primary	0	16	N/A	0.3
	Reoperation	7	52		
Smoking status	Never smoker	3	30	0.9 (0.2; 4.2)	>0.5
	Ever smoker	4	35		
Pseudarthrosis present at operation	Yes	2	13	1.7 (0.3; 9.7)	>0.5
	No	5	55		

Among surgical variables, fusion construct crossing 2 spine junctions (thoracolumbar and lumbosacral) was found to be a risk factor for RF with an OR of 9.1 (95 % CI: 1.0; 80.0, *P* = 0.05), Table 3. A few other surgical variables having relatively high risk showed only borderline statistical significance (0.05 > *P* < 0.2), in particular: osteotomy type - PSO vs. SPO (*OR* = 7.2, 95 % CI: 0.8; 62.7); fusion to sacrum (*OR* = N/A, *P* = 0.1); fusion to pelvis (*OR* = 3.2, 95 % CI: 0.70; 15.6); and ≥8 levels fused vs. <8 levels fused (*OR* = 3.8, 95 % CI: 0.4; 33.4), Table 3. Surgical variables that did not show significant association with RF were primary surgeon, osteotomy location, use of navigation systems (navigation vs. non-navigation), and presence of interbody support postop.

**Table 3:** Association rod fracture risk with surgical and instrumentation variables

Confounders	Subgroups	Rod(s) fracture		Odds ratio (95 % Cl: min; max)	P(*χ* ^2^)
		Yes	No		
Osteotomy by type	Pedicle subtraction	6	31	7.2 (0.8; 62.7)	0.1
	Smith-Peterson	1	37		
Osteotomy by location	Thoracolumbar junction	3	17	2.3 (0.5; 11.1)	0.3
	Other	4	51		
Use of navigation	Yes	7	62	N/A	>0.5
	No	0	5		
Use of cement	Yes	0	16	N/A	0.3
	No	7	48		
Screw/rod manufacturer	Device Company 1	3	29	0.9 (0.2; 4.3)	>0.5
	Other	4	34		
Pre-contoured rods	Yes	1	24	0.3 (0.03; 3.8)	0.4
	No	3	14		
Material of rods	Titanium	5	40	1.2 (0.06; 25.9)	>0.5
	Other	0	4		
Diameter of rods	6 mm	3	29	0.7 (0.1; 3.8)	>0.5
	Other	3	20		
Type of screws	Polyaxial	5	36	N/A	N/A
	Monoaxial	0	0		
Screw density	Incomplete^a^	4	27	2.0 (0.4; 9.8)	0.4
	Complete	3	41		
Connectors	Standard	6	48	2.5 (0.3; 22.1)	0.4
	Other	1	20		
Interbody support	Yes	4	45	0.7 (0.1; 3.2)	>0.5
	No	3	22		
Sagittal rod contour	>60°	5	19	10.0 (1.1; 95.1)	0.04
	≤60°	2	49		
Crosslinks	≥2	3	13	3.2 (0.6; 15.9)	0.2
	0–1	4	55		
Domino and/or parallel connectors^b^	Yes	4	8	10.0 (1.9; 53.1)	0.01
	No	3	60		
Number of fused levels^c^	≥8	6	41	3.8 (0.4; 33.4)	0.2
	8	1	26		
Number of crossing junctions	2	6	27	9.1 (1.0; 80.0)	0.05
	0–1	1	41		
Fusion to sacrum	Yes	7	38	N/A	0.1
	No	0	30		
Fusion to pelvis	Yes	4	20	3.2 (0.7; 15.6)	0.2
	No	3	48		

^a^Missing one or more pedicle screws at available locations along construct. ^b^Presence of domino and/or parallel-connectors at date of rod fracture. ^c^Including levels when connecting to prior instrumentation

Instrumentation variables provided two risk factors meeting statistical significance for RF: sagittal rod contour angle >60° (*OR* = 10.0, 95 % Cl: 1.1; 95.1, *P* = 0.04); and presence of domino and/or parallel connectors at date of fracture (*OR* = 10.0,95 % Cl: 1.9; 53.1, *P* = 0.01), Table 3. One instrumentation variable having relatively high OR showed only borderline statistical significance (0.05 > P < 0.2), ≥2 crosslinks vs. <2 (*OR* = 3.2, 95 % CI: 0.6; 15.9). Instrumentation variables not meeting significance included screw company, rod company, cement use, pre-contoured vs. straight rods, rod material, rod size, screw density, standard connectors vs. presence of other connectors, Table 3.

Only one postoperative variable met statistical significance and held the highest OR of the entire study: postoperative presence of pseudarthrosis at ≥1 year follow-up (*OR* = 28.9, Cl: 4.4; 191.7, *P* < 0.001), Table 4. No statistically significant association with RF was found for postoperative spinopelvic parameters such as: sagittal imbalance, coronal imbalance, lumbar lordosis, thoracic kyphosis, pelvic incidence, pelvic tilt, PI-LL, and coronal Cobb angle, Table 4.

**Table 4:** Association of rod fracture risk with postoperative characteristics

Confounders	Subgroups	Rod(s) fracture		Odds ratio (95 % Cl: min; max)	P(*χ* ^2^)
		Yes	No		
Postoperative sagittal imbalance	≥50 mm	3	22	1.5 (0.3; 8.1)	>0.5
	<50 mm	3	33		
Postoperative coronal imbalance	≥30 mm	2	16	1.2 (0.2; 7.1)	>0.5
	<30 mm	4	38		
Postoperative lumbar lordosis (LL)	<49°	4	21	2.5 (0.5; 12.4)	0.3
	≥49°	3	40		
Postoperative thoracic kyphosis	≥40°	3	33	0.5 (0.1; 2.6)	0.5
	<40°	4	23		
Postoperative pelvic incidence (PI)	<42°	2	11	1.8 (0.3; 11.0)	>0.5
	≥42°	4	39		
Postoperative PI-LL	>10°	1	16	0.4 (0.04; 3.3)	0.4
	≤10°	5	28		
Postoperative pelvic tilt	≤20	4	27	1.9 (0.3; 11.4)	0.5
	>20	2	26		
Postoperative coronal Cobb angle	<25°	7	54	N/A	0.4
	≥25°	0	14		
Pseudarthrosis at ≥1 year follow-up	Yes	4	3	28.9 (4.4; 191.7)	<0.001
	No	3	65		

### Risk factors analysis: clinically significant rod fractures

When the same variables were assessed in the 4 CSRFs, only two statistically significant risk factors were noted: fusion to pelvis (*P* = 0.05) and pseudarthrosis at ≥1 year follow-up (*OR* = 50.3, CI: 4.2; 598.8, *P* < 0.01), Table 5. Of note, multiple other factors including risk factors noted for all rod fractures held increased risk with borderline statistical significance (0.05 > *P* < 0.2) including: fusion construct crossing 2 spine junctions (OR = 4.1, CI: 0.4; 41.4, *P* = 0.2), sagittal rod contour >60° (*OR* = 7.1, CI: 0.7; 72.7, *P* = 0.1), presence of domino and/or parallel connectors at date of fracture (*OR* = 6.1, CI: 0.8; 48.4, *P* = 0.1), incomplete screw density (*OR* = 4.6, CI: 0.5; 46.5, *P* = 0.2), two or more crosslinks (*OR* = 4.1, CI: 0.5; 31.5, *P* = 02), and fusion to sacrum (*P* = 0.2), Table 5.

**Table 5:** Association of CSRF risk with variables showing increased risk with *P* ≤ 0.2

Confounders	Subgroups	CSRF fracture		Odds ratio (95 % Cl: min; max)	P(*χ* ^2^)
		Yes	No		
Screw density	Incomplete^a^	3	28	4.6 (0.5; 46.5)	0.2
	Complete	1	43		
Sagittal rod contour	>60°	3	21	7.1 (0.7; 72.7)	0.1
	≤60°	1	50		
Crosslinks	≥2	2	14	4.1 (0.5; 31.5)	0.2
	0–1	2	57		
Domino and/or parallel connectors^a^	Yes	2	10	6.1 (0.8; 48.4)	0.1
	No	2	61		
Number of crossing junctions	2	3	30	4.1 (0.4; 41.4)	0.2
	0–1	1	41		
Fusion to sacrum	Yes	4	41	N/A	0.2
	No	0	30		
Fusion to pelvis	Yes	4	20	N/A	0.05
	No	0	51		
Pseudarthorsis at	Yes	3	4	50.3 (4.2; 598.8)	<0.01
≥1 year follow-up	No	1	67		

^a^Missing one or more pedicle screws at available locations along construct

## Discussion

This study is a case-series of 75 consecutive ASD patients assessed for incidence and risk factors for RF and CSRF after instrumented posterior fusion and osteotomy (SPO and PSO). Total incidence of RF was 9.3 % with 16.2 % after PSO and 2.6 % after SPO. Clinically significant total rod fracture rate was 5.3 % and post-PSO rate was 8.1 %. Following risk factor analysis, statistically significant risk factors for RF were obtained: fusion construct crossing 2 spine junctions, sagittal rod contour >60°, presence of dominos and/or parallel connectors at date of fracture, and pseudarthrosis at ≥1 year follow-up. Statistically significant risk factors for the clinically significant subset were fusion to pelvis and pseudarthrosis at ≥1 year follow-up. Interestingly, most variables with increased risk, including those meeting statistical significance or borderline significance, were similar between total RF group and CSRF subset. However, due to the low number of only 4 CSRFs, most variables only met borderline significance.

Prior studies have reported a variable incidence of rod fracture after PSO, with two the largest studies to date reporting an incidence of 22 % for all rod fractures and 15.8 % for symptomatic rod fracture [18, 19]. Our overall post-PSO RF incidence of 16.2 % is comparable to what has been previously described, but our post-PSO CSRF rate of 8.1 % is approximately 1/2 the rate of what has been previous reported [18]. However, prior rates cannot be perfectly compared to our rates due to differences in study design including inclusion/exclusion criteria, for example prior study inclusion of ≥5 fusion levels vs. current study inclusion of ≥3 fusion levels. Further, we hypothesize that our lower rate may be underestimated due to minimum follow-up of 1 year in some of these patients, or due to differences in surgical technique and instrumentation trends at our institution. Several institutional trends associated with decreased rod strain include use of polyaxial screws (100 %) and use of anterior support including interbody fusion in a majority of our patients. A recent finite element analysis of a PSO model comparing mono-axial vs. poly-axial screws showed rod contour affected the location of bending moments and stress [34]. Anterior support below the PSO level reduced bending moments along the rod (−26 %) [34]. The lower incidence of CSRF could also be explained by patient demographic (Colorado population, patient selection) and surgeon expertise.

Our study found no significant impact of patient demographics or baseline characteristics on the RF or CSRF risk. It has been shown recently that patients with RF had higher BMI and age than those without RF [19]. Our results may differ based on the lower statistical power of our study or differences in design including: multicenter vs. single center and inclusion/exclusion criteria.

Surgical variable risk factors meeting statistical or borderline significance for RF and CSRF included: fusion constructs crossing two junctions and fusion to the pelvis. In prior studies, fusion across junctional regions of the spine, lumbosacral or thoraco-lumbar has been shown to be a risk factor for complications and increased strain on instrumentation [9, 14, 30, 32]. However, pelvic fixation alone has not been previously described as having association with RF. We propose that the association between CSRF and pelvic fixation could be the result of a few possible underlying etiologies. First, pelvic fixation may be a confounder to fusion across the lumbosacral junction of the spine, as pelvic fixation can only be present in those with fusion across the lumbosacral junction. Supporting evidence showed a high risk, with borderline significance, toward a greater proportion of RF and CSRF subjects with fusion to the sacrum compared to non-CSRF subjects. Alternatively, pelvic fixation alone may increase rod strain as pelvic fixation has been shown to increase overall spinal construct stiffness, but may inflict pinpoint strain on the rods [35]. Supporting evidence is seen with multiple rods fracturing near or at iliac bolt connector. Lastly, an extremely unbalanced spine requiring additional support can be a factor necessitating pelvic fixation and thus contributing to rod stress. Therefore, a spine with a high degree of imbalance at baseline, rather than the pelvic fixation alone, may explain the association between CSRF and pelvic fixation. Supporting literature found increased risk for RF with increased baseline sagittal imbalance and increased SVA correction [19]. Another surgical variable showing increased risk for RF, but only meeting borderline significance, was osteotomy type - PSO vs. SPO. Prior studies haven’t quantified this risk. However, PSOs have a couple factors that may increase rod strain including absence of pedicle screws at PSO level and greater degree of correction obtained with PSO vs. SPO.

Our findings support previous experimental data that excessive rod contouring causes notching and internal strain that decreases rod fatigue strength and increases risk of RF [20–24, 26, 27, 36]. In particular: sagittal rod bend >60° and presence of dominos and/or parallel-connectors were found to be statistically significant instrumentation risk factors for RF. These two variables also showed increased risk, but only held borderline significance in the CSRF subset. Over half of fractured rods had failed at the apex of rod bend with rod bends averaging over 60°. One CSRF occurred at an area previously noted in the operative note that the rod was “bent out of way” and subsequently bent back into a new iliac bolt. Two RF subjects had rod fractures located at or near a domino connector. Presence of domino or parallel connectors was evaluated at date of fracture, to account for a subsequent operation that may have used these connections to connect to original instrumentation. This finding is important because revision surgery and connection to prior instrumentation often calls for re-contouring of the end of the rod to match new instrumentation. Other variables associated with increased risk but only meeting borderline statistical significance included ≥2 crosslinks vs. <2 crosslinks (RF and CSRF) and screw density, complete vs. incomplete (CSRF only). Quantified risk pertaining to these factors has not been published in prior studies.

Pseudarthrosis was revealed in over half of RF subjects and ¾ of CSRF subjects, showing statistically significant association with rod fracture. This result provides statistic confirmation of previous findings, and can be explained by effect of cyclic loading at a non-fused segment allowing micro-movements to increase construct strain and risk of instrumentation failure [4, 6, 14, 18].

Previous studies suggested that residual postoperative sagittal imbalance may be associated with risk of instrumentation failure; however, these studies are without statistical confirmation [18, 29]. A recent study on RF found the amount of SVA correction, rather than postoperative SVA was a significant risk for RF [19]. The risk analysis performed in our study did not reveal significant association between postoperative spinopelvic parameters and RF/CSRF. This finding could simply be contributed to our small cohort, or may signify that differences in surgical technique and instrumentation may protect against under correction. Of note, 40 % of non-RF subjects in our study had postoperative sagittal imbalance of >5 cm and didn’t experience a rod fracture. Only 3 of the 25 total subjects with postoperative sagittal imbalance developed RF during the follow-up period.

Our study had several limitations, the main of which are: retrospective design with the inherent risk of selection bias; and relatively small sample size likely cause underestimation of significance for a few studied risk factors. Further, missing data including the type of interbody device and bone graft and rod characteristics in multiple patients should be regarded as a limitation. Therefore, the presented results should be viewed as preliminary basis for further prospective studies with a larger cohort.

Based on these preliminary findings found in our retrospective review case series, it may be reasonable for clinicians to try and eliminate or reduce these risk factors for rod fracture in their ASD patients to possibly decrease the risk of rod fracture. With consideration of the limitations in our study, incidence of rod fracture may be decreased by: 1) pursuing all means to avoid post-operative pseudarthrosis 2) use the smallest sagittal rod contour possible while providing adequate correction, in particular <60° 3) decrease use of domino and/or parallel rod connectors when possible 4) if unnecessary, do not implement pelvic fixation. Finally, patients with radiographic evidence of pseudarthrosis after 1 year post-operatively should be regarded as having increased risk of rod fracture and may require more careful observation.

## Conclusions

1. The incidence of rod fracture varies depending on impact of different confounders. This study found incidence rates of 9.3 % for all RFs, 16.2 % for all RF after PSO, 2.6 % for all RF after SPO, 5.3 % for all CSRFs, and 8.1 % for CSRF after PSO.
2. The risk of RF and CSRF is mainly determined by surgical and instrumentation factors rather than baseline patient demographics or postoperative spinopelvic parameters.
3. The following factors have significant association with the risk of rod fractures after posterior instrumented adult spinal deformity correction with osteotomy: pseudarthrosis at ≥1 year follow-up, sagittal rod contour >60°, presence of dominos and/or parallel connectors at date of fracture, and fusion construct crossing 2 junctions. Risk factors for clinically significant subset of rod fractures meeting statistical significance includes pseudarthrosis at ≥1 year follow-up and pelvic fixation.
